# Non-genomic action of resveratrol on androgen and oestrogen receptors in prostate cancer: modulation of the phosphoinositide 3-kinase pathway

**DOI:** 10.1038/sj.bjc.6603755

**Published:** 2007-05-08

**Authors:** D A Benitez, E Pozo-Guisado, M Clementi, E Castellón, P M Fernandez-Salguero

**Affiliations:** 1 Laboratorio de Andrología Celular y Molecular, PDFB, ICBM, Facultad de Medicina, Universidad de Chile, P.O. Box 70005, Santiago de Chile, Chile; 2 Departamento de Bioquímica y Biología Molecular, Facultad de Ciencias, Universidad de Extremadura, 06080, Badajoz, Spain

**Keywords:** resveratrol, prostate cancer, PI3K pathway, androgen receptor, oestrogen receptor, GSK-3

## Abstract

Prostate cancer represents a major concern in human oncology and the phytoalexin resveratrol (RES) inhibits growth and proliferation of prostate cancer cells through the induction of apoptosis. In addition, previous data indicate that in oestrogen-responsive human breast cancer cells, RES induces apoptosis by inhibition of the phosphoinositide-3-kinase (PI3K) pathway. Here, using androgen receptor (AR)-positive LNCaP and oestrogen receptor alpha (ER*α*)-expressing PC-3 prostate tumour cells, we have analysed whether the antiproliferative activity of RES takes place by inhibition of the AR- or ER*α*-dependent PI3K pathway. Although RES treatment (up to 150 *μ*M) decreased AR and ER*α* protein levels, it did not affect AR and ER*α* interaction with p85-PI3K. Immunoprecipitation and kinase assays showed that RES inhibited AR- and ER*α*-dependent PI3K activities in LNCaP and PC-3, respectively. Consistently, lower PI3K activities correlated with decreased phosphorylation of downstream targets protein kinase B/AKT (PKB/AKT) and glycogen synthase kinase-3 (GSK-3). GSK-3 dephosphorylation could be responsible for the decreased cyclin D1 levels observed in both cell lines. Importantly, RES markedly decreased PKB/AKT phosphorylation in primary cultures from human prostate tumours, suggesting that the mechanism proposed here could take place *in vivo*. Thus, RES could have antitumoral activity in androgen-sensitive and androgen-non-sensitive human prostate tumours by inhibiting survival pathways such as that mediated by PI3K.

Trans-resveratrol (3,4′,5-trihydroxystilbene, RES) is a phytoalexin that has gained considerable interest because of its ability to inhibit cell proliferation in tumour cells of different origin (Ahmad *et al*, 2001; Dorrie *et al*, 2001; Joe *et al*, 2002; Pozo-Guisado *et al*, 2002). *In vivo*, several studies have shown that RES inhibits tumour growth in xenograft mouse models for skin (Jang *et al*, 1997), mammary gland (Banerjee *et al*, 2002; Whitsett *et al*, 2006) and colorectal (Sengottuvelan *et al*, 2006) cancer. Particularly, interesting is the effect of RES as a selective apoptotic inducer in tumour cells that respond to steroid hormones. Thus, previous studies have revealed that RES triggered apoptosis in oestrogen receptor alpha (ER*α*)-positive MCF-7 but not in ER*α*-negative MDA-MB-231 human breast tumour cells. These specific effects were associated to cell type-specific regulation of proteins controlling the G1/S (cyclin D1, cyclin E) and G2/M (cyclin B, cdc2^p34^) transitions of the cell cycle (Lu and Serrero, 1999; Pozo-Guisado *et al*, 2002). The ER*α* is not only a transcriptional regulator in the cell nucleus but also a cytosolic intermediate in the survival pathway regulated by phosphoinositide-3-kinase (PI3K) (Simoncini *et al*, 2000; Marquez and Pietras, 2001; Cantley, 2002). Based on these observations, we previously reported that the apoptosis induced by RES in oestrogen-dependent MCF-7 cells was mediated by inhibition of the ER*α*-associated PI3K activity (Pozo-Guisado *et al*, 2004). Further, this effect involved a caspase-independent mechanism with downregulation of Bcl-2 and NF-*κ*B (Pozo-Guisado *et al*, 2005). Several studies reported that RES has a complex effect in oestrogen-responsive cells, acting as antagonist or agonist for the ER*α* in different cell types and cellular contexts (Gehm *et al*, 1997; Lu and Serrero, 1999; Basly *et al*, 2000; Bowers *et al*, 2000; Bhat *et al*, 2001; Levenson *et al*, 2003; Pozo-Guisado *et al*, 2004).

Among PI3Ks, those belonging to the IA class are composed of a regulatory subunit (p85*α*) and a catalytic peptide (p110). Functionally, p85*α* links membrane-bound growth factor receptors to p110, which synthesises lipid intermediates such as phosphatidylinositol-3,4,5-triphosphate (PI(3,4,5)P_3_) (Cantley, 2002). PI(3,4,5)P_3_ functions as a docking molecule for the membrane localisation of proteins harbouring pleckstrin homology domains such as protein kinase B/AKT (PKB/AKT) (Stephens *et al*, 1998). One of relevant target proteins of PKB/AKT is glycogen synthase kinase-3 (GSK-3), which becomes inactivated by PKB/AKT-dependent phosphorylation (Cross *et al*, 1995). In unstimulated cells (e.g. low PKB/AKT activity), active GSK-3 phosphorylates proteins such as cyclin D1, c-myc and glycogen synthase, thus promoting their degradation and leading to downregulation of the cell cycle. Under conditions of PI3K activation, PKB/AKT phosphorylates and inactivates GSK-3, which results in increased levels of metabolic and cell cycle regulatory proteins that will drive the G1/S transition (Scheid and Woodgett, 2001; Scheid *et al*, 2002). As over-activation of the PI3K pathway has been linked to human disease, a better knowledge of its molecular intermediates in different cell types could help to find novel targets and to characterise new therapeutic molecules against human cancer (Scheid and Woodgett, 2001).

The effects of RES on different steroid hormone-responsive tumour cells appear to follow a common mechanism. Similar to that observed in ER*α*-positive MCF-7 breast tumour cells, RES inhibited DNA synthesis and modulated cell cycle progression in androgen receptor (AR)-positive LNCaP but not in AR-negative DU145 human prostate tumour cells (Hsieh and Wu, 1999; Kuwajerwala *et al*, 2002). Recent studies have also shown that AR status could cause a differential effect of RES on cell cycle regulation at the G1/S transition in LNCaP and PC-3 cells (Benitez *et al*, 2007). Moreover, RES modulated the transcriptional activity of the AR in LNCaP prostate cancer cells (Mitchell *et al*, 1999; Gao *et al*, 2004), inducing changes in gene expression (Narayanan *et al*, 2003) that were similar to those observed after inhibiting the transcriptional activity of the ER*α* in MCF-7 breast tumour cells (Pozo-Guisado *et al*, 2004). Finally, the interaction of steroid hormone receptors with PI3K is not exclusive of the ER*α*, as the AR can also activate this kinase by forming a complex with p85 and Src (Sun *et al*, 2003). In a recent study, Aziz *et al* (2006) reported that RES decreased PKB/AKT phosphorylation in LNCaP cells and associated this effect with the induction of apoptosis by the intrinsic mitochondrial pathway. Interestingly, a similar correlation has been proposed for apoptosis induction by RES in MCF-7 breast cancer cells, in which inhibition of PI3K resulted in lower PKB/AKT activity, NF-*κ*B inhibition and Bcl-2 downregulation (Pozo-Guisado *et al*, 2004; Pozo-Guisado *et al*, 2005).

Based on these previous results, it appears that RES triggers specific mechanisms of apoptosis in a cell type selective manner in steroid hormone-responsive breast and prostate cancer cells. In this work, we have used LNCaP (AR positive, ER*α* negative) and PC-3 (AR negative, ER*α* positive) prostate tumour cells to address the mechanism through which RES modulates the AR- and ER*α*-associated PI3K activity. We have found that RES inhibited, in a concentration-dependent manner, AR- and ER*α*-dependent PI3K activity in LNCaP and PC-3, respectively. PI3K inhibition correlated with PKB/AKT and GSK-3 phosphorylation and with decreased cyclin D1 levels. Further, RES also inhibited PKB/AKT phosphorylation in cultured cells from primary human prostate tumours. We suggest that AR and ER*α*-associated PI3K could represent novel target proteins for the antitumoral activity of RES in human prostate tumours, establishing a common mechanism with other hormone-dependent cancers such as breast.

## MATERIALS AND METHODS

### Reagents

RES, dihydrotestosterone (DHT), 17*β*-oestradiol (E2), ATP, L-*α*-phosphatidylinositol (PI) and DMSO were purchased from Sigma-Aldrich (St Louis, MO, USA). ICI 182,780 and Bicalutamide (Bic) were a generous gift from Zeneca Pharmaceuticals (Macclesfield, UK). The PI3K inhibitor LY294002 was used at 20 *μ*M and was obtained from Calbiochem (La Jolla, CA, USA). Dulbecco's Modified Eagle Medium (DMEM) and DMEM : Nutrient mixture F-12 (Ham) (1 : 1) (DMEM/F-12) were from Invitrogen (Carlsbad, CA, USA). Foetal bovine serum (FBS) was from BioWhittaker (East Rutherford, NJ, USA) and was heat inactivated before use. Protein A/G plus agarose was from Santa Cruz Biotechnology (Santa Cruz, CA, USA). Antibodies used in this study were: PKB/AKT1/2 (559028), phospho-PKB/AKT (550747) and p85-PI3-kinase (610045) from Becton-Dickinson (San Jose, CA, USA), p85-PI3-kinase (06497) from Upstate Biotechnology (Waltham, MA, USA), AR Ab-1, ER*α* Ab-10 (immunoprecipitation) and ER*α* Ab-16 (immunoblotting) from NeoMarkers (Fremont, CA, USA), *β*-actin (A2066) from Sigma-Aldrich and from MP-Biomedicals (Solon, OH, USA) (69100) and GSK-3 (9331) and p-PKB/AKT (4058) from Cell Signaling (Danvers, MA, USA). The antibody against phospho-GSK-3 was a generous gift from Dr Dario Alessi (University of Dundee, UK).

### Human prostate and breast cancer cells and prostate cancer primary cell cultures

The human tumour cell lines used in this study were purchased from the American Type Culture Collection (Manassas, VA, USA). Prostate cancer androgen-sensitive LNCaP and androgen-insensitive PC-3 cell lines were cultured in DMEM/F-12 supplemented with 10% heat-inactivated FBS, 100 units/ml penicillin G, 100 *μ*g ml^−1^ streptomycin and 30 *μ*g ml^−1^ amphotericin B. Breast cancer cell lines MCF-7 (oestrogen responsive) and MDA-MB-231 (oestrogen unresponsive) were grown in DMEM supplemented with 10% heat-inactivated FBS, 2 mM L-glutamine, 100 units ml^−1^ penicillin G, 100 *μ*g ml^−1^ streptomycin and 30 *μ*g ml^−1^ amphotericin B. Treatments with *trans*-RES (in DMSO) were carried out for 36 h with the addition of fresh RES and culture medium at 24 h. In experiments requiring steroid-free conditions, cells were maintained for 5 days in phenol red-free DMEM : F12 (1 : 1) supplemented with charcoal-stripped 10% FBS.

Human prostate cancer biopsies were obtained from patients scheduled for radical prostatectomy at the Clinical Hospital of the University of Chile. Informed consent was obtained from the patient's guardian and the experimental protocol approved by the ethics committee of the Institution. Primary cultures were established and characterised from seven prostate tumours as described (Castellon *et al*, 2005). Briefly, small tissue fragments were digested in an enzymatic mixture containing 2.5 mg ml^−1^ collagenase, 1 mg ml^−1^ hyaluronidase and 0.01 mg ml^−1^ deoxyribonuclease for 2–3 h at 37°C in a shaking water bath. The epithelial cell aggregates were washed and further digested in collagenase solution for another 8–12 h under the same conditions. The small aggregates of prostate cancer cells obtained at the end of the incubation were mechanically dispersed, washed and seeded in cell culture plates. During the first days, culture medium was supplemented with 5% FBS. To verify the tumoral phenotype of these primary cells, cultures were stained by immunocytochemistry for the malignant epithelial marker PCTA-1. Close to 90% of the cells were positive for PCTA-1, thus revealing their transformed status. Detailed description of the isolation, culturing and characterisation of these primary cultures have been published elsewhere (Sanchez *et al*, 2005; Castellon *et al*, 2006, 2005).

### Immunoprecipitation and associated PI3-kinase activity

Androgen- and oestrogen receptor-associated PI3K activity was determined in AR and ER*α* immunoprecipitates by measuring the *in vitro* phosphorylation of PI into L-*α*-phosphatidylinositol-3-phosphate as described (Pozo-Guisado *et al*, 2004). LNCaP and PC-3 cells growing in complete or steroids-depleted medium were treated for 36 h with RES and then lysed on ice for 15 min with 500 *μ*l IP buffer (20 mM Tris-HCl pH 7.4, 50 mM NaCl, 1% Nonidet P-40) containing 10 mM EDTA, 1 mM sodium orthovanadate, 50 mM NaF, 0.5 mM phenyl-methyl sulphonyl fluoride (PMSF) and 4 *μ*g ml^−1^ Complete protease inhibitor cocktail (Roche, Nutley, NJ, USA). Lysates were centrifuged at 15 000 g for 15 min at 4°C. Protein concentration was determined in the supernatants using the Coomassie Plus protein assay reagent (Pierce, Rockford, IL, USA) and bovine serum albumin as standard. One milligram of protein from freshly prepared extracts was used for each immunoprecipitation. AR and ER*α* were immunoprecipitated overnight at 4°C with 1 *μ*g anti-AR Ab-1 or 1.5 *μ*g anti-ER*α* Ab-10 antibodies, respectively. Next, 25 *μ*l of protein A/G plus-agarose beads were added and the samples incubated for an additional 1 h at 4°C. Beads were washed twice with each of the following buffers: buffer A (25 mM Tris-HCl pH 7.5, 1% Nonidet P-40, 0.1 mM sodium orthovanadate); buffer B (100 mM Tris-HCl pH 7.5, 0.1 mM sodium orthovanadate, 1 mM EDTA, 0.5 M LiCl); buffer C (25 mM Tris-HCl pH 7.5, 150 mM NaCl, 1 mM EDTA). To measure AR- or ER*α*-associated PI3K activity, 30 *μ*g PI (reconstituted and sonicated in 25 mM HEPES pH 7.5, 1 mM EDTA) were pre-incubated with the beads for 15 min at 4°C. Enzymatic reactions were performed at room temperature for 30 min in 15 mM HEPES pH 7.6, 10 mM MgCl_2_, 0.5 mM EGTA, 40 *μ*M nonlabelled ATP and 10 *μ*Ci [^32^P]-*γ*ATP (sp. act., 6000 Ci mmol^−1^). Proteins were then denatured by adding 400 *μ*l of a chloroform-methanol solution (1 : 2) in 1% HCl, plus 125 *μ*l chloroform and 125 *μ*l 10 mM HCl. Samples were centrifuged and the organic phase washed once with 500 *μ*l of methanol : 100 mM HCl (1 : 1) plus 2 mM EDTA. The organic phase was recovered, dried under nitrogen and resuspended in 30 *μ*l chloroform. Phosphorylated lipids were resolved by thin layer chromatography (TLC) using 60F254 silicagel plates (Merck, Whitehouse Station, NJ, USA) and a solution composed of chloroform : methanol : ammonia : water (120 : 94 : 4 : 23.2) as mobile phase. TLC plates were exposed in a Molecular Imager FX system (Bio-Rad Labs, Hercules, CA, USA) and analysed using the Quantity One software (Bio-Rad Labs). To determine the interaction between AR and ER*α* with PI3K, proteins immunoprecipitated by the Ab-1 or Ab-16 antibodies were analysed by SDS-PAGE and Western immunobloting using a p85/PI3K-specific antibody.

### SDS-PAGE and Western immunobloting

After treatment with RES, cells were washed with cold PBS and lysed in ice-cold lysis buffer (50 mM Tris-HCl pH 7.5, 2 mM EDTA, 2 mM EGTA, 10 mM *β*-glycerophosphate, 150 mM NaCl, 0.5% Nonidet P40, 1 mM PMSF, 1 mM NaF, 1 mM DTT, 1% *β*-mercaptoethanol and 4 *μ*g ml^−1^ Complete protease inhibitor cocktail (Roche, Nutley, NJ, USA). Lysates were centrifuged at 15 000 g for 15 min at 4°C and protein concentration determined in the supernatants using the Coomassie Plus protein assay reagent (Pierce, Rockford, IL, USA) and bovine serum albumin as standard. Fifteen micrograms of protein were mixed with SDS sample buffer, denatured and electrophoresed in 10 or 12% SDS-PAGE gels. Gels were transferred to nitrocellulose membranes by electroblotting and blocked for 2 h at room temperature in TBS-T (50 mM Tris-HCl pH 7.5, 150 mM NaCl, 0.2% Tween-20) containing 7% nonfat milk. Blots were sequentially incubated with the primary and secondary antibodies, washed in TBS-T and revealed using the Super-signal luminol substrate (Pierce, Rockford, IL, USA) and a chemiluminescence-imaging screen (Bio-Rad, Hercules, CA, USA). The screen was scanned using a Molecular Imager FX system from Bio-Rad (Hercules, CA, USA). For reprobing, blots were stripped by incubation in 100 mM Tris-HCl, pH 7.4, 100 mM *β*-mercaptoethanol and 2% SDS at 50°C for 30 min.

### Statistical analysis

Data are expressed as mean±s.e.m. Statistical comparison between treatments was carried out using GraphPad Prism 4.0 software (GraphPad, San Diego, CA, USA). One-way ANOVA followed by Dunn test were applied. ^*^P<0.05 and ^**^P<0.01.

## RESULTS

### RES treatment did not affect p85/PI3K levels but it decreased AR and ER*α* protein expression in LNCaP and PC-3 cells

**Figure 1:**
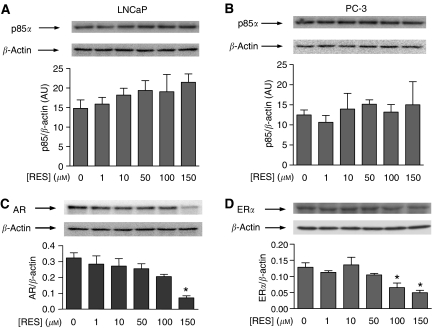
RES does not significantly affect p85/PI3K but decreases AR and ER*α* levels in LNCaP and PC-3 cells. LNCaP (**A**, **C**) and PC-3 (**B**, **D**) were left untreated (0, DMSO) or treated with 1, 10, 50, 100 or 150 *μ*M RES for 36 h. Total protein extracts were obtained and analysed for p85/PI3K (**A**, **B**), AR (**C**) or ER*α* (**D**) protein expression by Western immunobloting using specific antibodies. The level of *β*-actin was also determined to account for protein quantitation and equal loading. p85/PI3K, AR and ER*α* expression were quantitated and normalised by *β*-actin in at least three different cultures. Data shown are mean±s.e. The difference with respect to untreated cultures (DMSO) is significant at *P*<0.05 (^*^).

The main goal of this study was to analyse whether the antiproliferative activity of RES in human prostate cancer cells could be mediated by inhibition of the AR- and ER*α*-dependent PI3K pathways. The effect of RES on AR- and ER*α*-dependent signalling was analysed after 36 h of treatment, as no significant apoptosis was observed in short-term (e.g. 30 min) treatments (Benitez *et al*, 2007). Considering that the interaction between cytosolic steroid receptors (e.g. ER*α*) and PI3K presumably takes place through p85 (Simoncini *et al*, 2000), we first determined if RES treatment could decrease protein levels for p85/PI3K, AR and ER*α*. Concentrations of RES up to 150 *μ*M did not significantly decrease endogenous p85 protein in androgen-responsive LNCaP or in androgen-insensitive PC-3 cells (Figure 1A and B). Regarding AR status in LNCaP, RES treatment induced a concentration-dependent decrease in receptor levels that was more pronounced at the highest concentration used of 150 *μ*M (Figure 1C). In PC-3, an androgen-insensitive prostate cancer cell line not expressing AR but having ER*α* (Lau *et al*, 2000), RES treatment also produced a concentration-dependent reduction in ER*α* with a maximum effect at 100–150 *μ*M (Figure 1D). Thus, in our experimental conditions, RES did not significantly influence p85 but decreased the cellular levels of AR and ER*α*, an effect that could alter their likely interaction with the PI3K enzyme.

### AR and ER*α* interacted with p85/PI3K in LNCaP and PC-3 cells and RES did not affect such interactions

**Figure 2:**
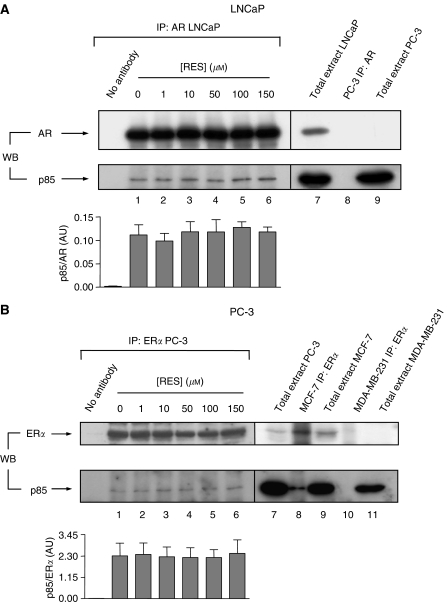
Steroid receptors AR and ER*α* interact with p85/PI3K in LNCaP and PC-3 prostate cancer cells and such interaction is not altered by RES treatment. LNCaP (**A**) and PC-3 (**B**), growing in complete medium, were treated with the indicated concentrations of RES for 36 h and 1 mg total cell extracts immunoprecipitated (IP) with anti-AR- or anti-ER*α*-specific antibodies, respectively. The amount of p85/PI3K associated to each receptor was determined in the immunoprecipitates by Western immunobloting (WB) (lower blots in **A** and **B**, lanes 1–6). For quantitation, immunoprecipitated p85 was normalised by the amount of AR (**A**) or ER*α* (**B**) at each concentration of RES in three different cultures (lanes 1–6). For LNCaP cells (**A**) total cell extract was used as a positive control for AR and p85 expression (lane 7), whereas negative controls for this cell line included AR immunoprecipitation (lane 8) and total extract (lane 9) from androgen-insensitive PC-3 cells. For PC-3 (**B**), positive controls for ER*α* and p85 expression included total cell extracts from this cell line (lane 7) and from human breast tumour MCF-7 cells (lane 9); for ER*α* association to p85, immunoprecipitations were carried out in oestrogen-responsive MCF-7 (lower blot in **B**, lane 8). Negative controls were also included for ER*α* expression (lane 11) and for its interaction with p85 (lane 10) using oestrogen-unresponsive human breast tumour MDA-MB-231 cells. As an additional negative control, immunoprecipitations were carried out in the absence of AR or ER*α* antibodies (no antibody in **A** and **B**).

It was shown that the cytosolic ER*α* interacted with PI3K in cancer cells (Simoncini *et al*, 2000; Pozo-Guisado *et al*, 2004). To determine if a similar mechanism was taking place in LNCaP and PC-3 prostate cancer cells, co-immunoprecipitation experiments were performed using specific antibodies for AR and ER*α* (Figure 2). In the absence of RES (basal cell conditions), the AR antibody was able to immunoprecipitate p85 in LNCaP (Figure 2A, lower blot, lane 1 and graph) and the ER*α* antibody to immunoprecipitate this kinase in PC-3 (Figure 2B, lower blot, lane 1 and graph). The AR–p85 complexes were specific, as they were not recovered in AR-negative PC-3 cells (Figure 2A, lower panel, lane 8). Similarly, the ER*α*–p85 complexes found in PC-3 were specific, as they could be observed in ER*α*-positive MCF-7 but not in ER*α*-negative MDA-MB-231 breast tumour cells (Figure 2B, lower blot, lanes 8 and 10, respectively). Reasonably, the levels of p85 bound to AR in LNCaP (Figure 2A, lower blot, compare lanes 1, 7 and 9) or to ER*α* in PC-3 cells (Figure 2B, lower blot, compare lanes 1, 7, 9 and 11) were significantly lower than the total cellular content of p85, indicating that only an small fraction of kinase was interacting with these steroid receptors. Interestingly, treatment with RES up to 150 *μ*M did not significantly affect p85 interaction with AR in LNCaP or with ER*α* in PC-3 cells (Figure 2A and B, lower blots, lanes 1–6), suggesting that the decrease in AR and ER*α* protein levels at high concentrations of RES (Figure 1C and D) was not a limiting factor in the formation of complexes between these steroid receptors and PI3K.

### RES modulated the AR- and ER*α*-associated PI3K activity in LNCaP and PC-3 prostate tumour cells

**Figure 3:**
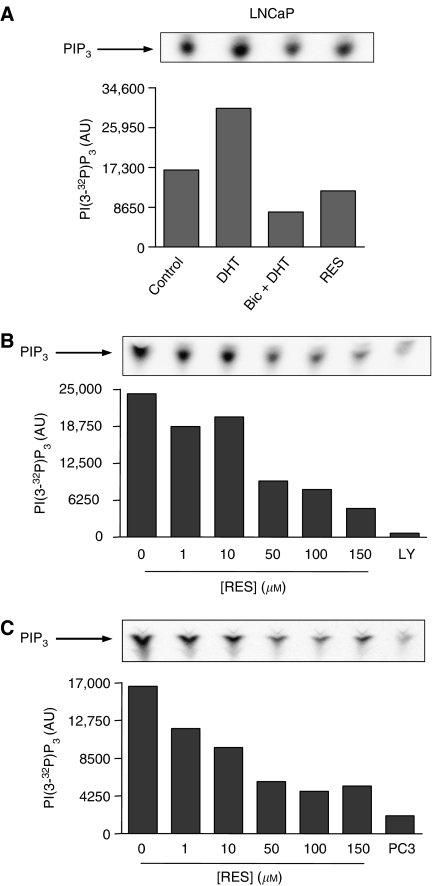
The AR-associated PI3K activity is modulated by RES in androgen-sensitive LNCaP cells. (**A**) LNCaP cells were cultured for 5 days in steroid-depleted medium and then treated with DMSO (control), 10^−9^ M of the AR agonist DHT (30 min), 10^−9^ M DHT (30 min) plus 10^−4^ M of the AR antagonist Bic (30 min) or 150 *μ*M RES (30 min). Aliquots of 1 mg total cell extract were immunoprecipitated with an AR-specific antibody and PI3K activity determined in the immunoprecipitates using PI as substrate. (**B**) PI3K activity was determined in AR immunoprecipitates obtained from cultures grown in steroid-depleted medium and left untreated (DMSO) or treated with 1, 10, 50, 100 or 150 *μ*M RES for 36 h. Some cultures were treated with 20 *μ*M of the PI3K inhibitor LY294002 (LY). (**C**) PI3K activity was also determined in AR immunoprecipitates from cultures grown in complete medium and treated with 0 (DMSO), 1, 10, 50, 100 or 150 *μ*M RES. Androgen-insensitive PC-3 cells (PC3) were used as negative control. A representative experiment out of two is shown.

Previous studies have shown that ER*α* activation increased PI3K signalling in endothelial cells (Simoncini *et al*, 2000) and that RES has oestrogenic and anti-oestrogenic activities (Lu and Serrero, 1999; Bowers *et al*, 2000; Bhat and Pezzuto, 2001; Bhat *et al*, 2001) that could help explain its effects on the ER*α*-associated PI3K activity in MCF-7 breast tumour cells (Pozo-Guisado *et al*, 2004). Based on these results, and as p85/PI3K interacted with AR and ER*α* in LNCaP and PC-3 cells, we have analysed if this phytoalexin could inhibit the PI3K activity associated to these receptors. In basal LNCaP, PI3K activity could be detected in AR immunoprecipitates (Figure 3A, control), indicating that this pathway was active under normal culture conditions. This kinase activity was dependent not only on the interaction between AR and PI3K but also on the activity of the receptor, as it could be increased by treatment with the AR ligand DHT and decreased below basal levels by co-treatment with DHT plus the antagonist Bic (Figure 3A, Bic+DHT). Further, treatment with 150 *μ*M RES for 30 min induced a reproducible decrease in AR-associated PI3K activity (Figure 3A, RES). We then determined the effect of increasing concentrations of RES for 36 h on the AR-dependent PI3K activity in LNCaP cells growing in steroids-depleted medium. RES induced a concentration-dependent inhibition of PI3K activity that reached very low levels at 150 *μ*M (Figure 3B). This kinase activity was produced by PI3K, as it could be blocked by its specific antagonist LY294002 (Figure 3B, LY). A similar titration curve was obtained in LNCaP cells cultured in complete medium (Figure 3C), suggesting that FBS components did not significantly affect the AR-associated PI3K activity. Androgen-insensitive PC-3 cells, as expected, had only a residual level of AR-dependent PI3K activity (Figure 3C, PC3).

**Figure 4:**
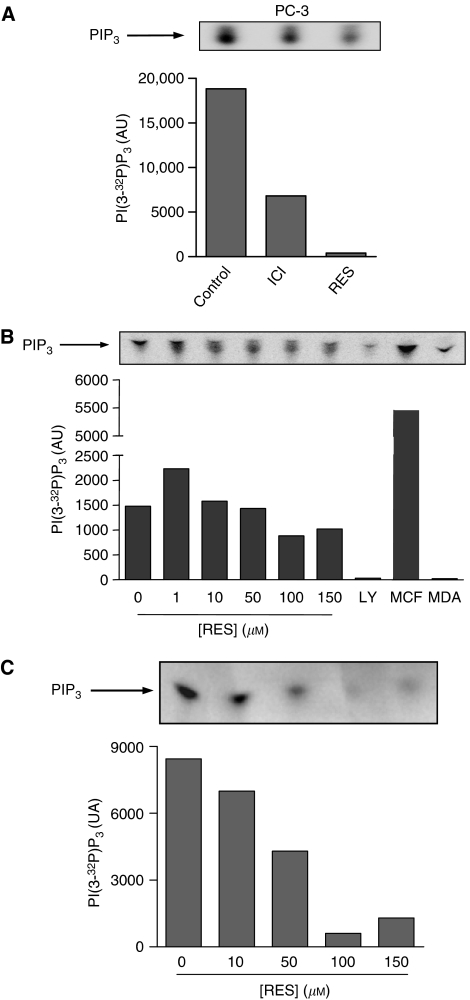
RES modulates the ER*α*-associated PI3K activity in androgen-insensitive PC-3 cells. (**A**) PC-3 cells were cultured in steroid-depleted medium for 5 days and then treated with DMSO (control), 1 *μ*M of the ER*α* antagonist ICI 182,780 (30 min) or 150 *μ*M RES (30 min). Total cell extracts were prepared and 1 mg protein immunoprecipitated with an ER*α*-specific antibody. ER*α*-associated PI3K activity was determined in the immunoprecipitates using PI as substrate. (**B**) PC-3 cells growing in steroid depleted medium for 5 days were left untreated (DMSO) or treated with 1, 10, 50, 100 or 150 *μ*M RES for 36 h and PI3K activity determined in ER*α* immunoprecipitates. Cells were also treated with 20 *μ*M of the PI3K inhibitor LY294002 (LY). As positive and negative controls, experiments were performed in ER*α* immunoprecipitates from ER*α*-positive MCF-7 and ER*α*-negative MDA-MB-231 breast cancer cells, respectively. (**C**) PC-3 cells were grown in complete medium and left untreated (DMSO) or treated with 10, 50, 100 or 150 *μ*M RES for 36 h. PI3K activity was determined in ER*α* immunoprecipitates using PI as substrate. A representative experiment from two is shown.

With respect to ER*α*-expressing PC-3 cells (Figure 4), a constitutive level of PI3K activity was detected in ER*α* immunoprecipitates, indicating that oestrogens could regulate the PI3K pathway in androgen-insensitive prostate tumour cells (Figure 4A, control). In agreement, the specific ER*α* antagonist ICI 182,780 blocked such induction (Figure 4A, ICI). Treatment with 150 *μ*M RES for 30 min also inhibited the ER*α*-associated PI3K activity (Figure 4A, RES), suggesting that this chemopreventive molecule had anti-oestrogenic activity in PC-3 cells. In addition, RES treatment for 36 h produced a concentration-dependent inhibition of the ER*α*-dependent PI3K activity in steroids-depleted medium (Figure 4B). This kinase activity was due to PI3K, as it was markedly inhibited by its specific antagonist LY294002 and ER*α*-dependent, because it was present in ER*α*-positive MCF-7 but not in ER*α*-negative MDA-MB-231 breast cancer cells (Figure 4B). In complete medium, RES treatment also inhibited the ER*α*-dependent PI3K activity in a concentration-dependent manner (Figure 4C). Thus, LNCaP and PC-3 cells had an endogenous steroid receptor-dependent PI3K activity that could be modulated by RES through inhibition of AR and ER*α* receptors.

### RES modulated PKB/AKT and GSK-3 phosphorylation and cyclin D1 levels in LNCaP and PC-3 cells with a pattern similar to that of PI3K activity

**Figure 5:**
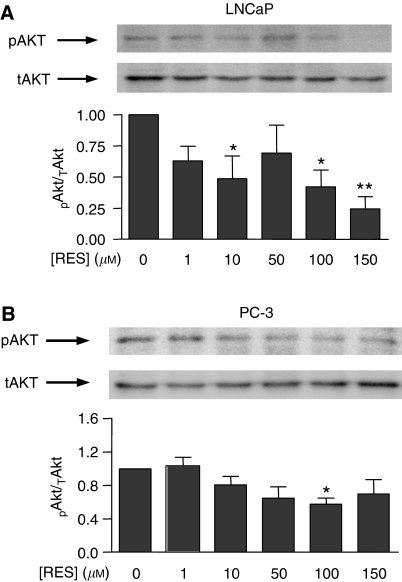
RES inhibition of the PI3K activity in LNCaP and PC-3 cells resulted in decreased PKB/AKT phosphorylation. LNCaP (**A**) and PC-3 (**B**) cells were left untreated (DMSO) or treated with 1, 10, 50, 100 or 150 *μ*M RES for 36 h in complete medium. Protein extracts were analysed by Western immunobloting to detect the phosphorylated (pAKT) and the total PKB/AKT protein (tAKT). Data were quantitated by normalising pPKB/AKT by tPKB/AKT (pAKT/tAKT) and the results plotted against the concentration of RES. Data shown are mean±s.e. from three different cultures. The differences with respect to control cultures (DMSO) are significant at *P*<0.05 (^*^) or *P*<0.01 (^**^).

**Figure 6:**
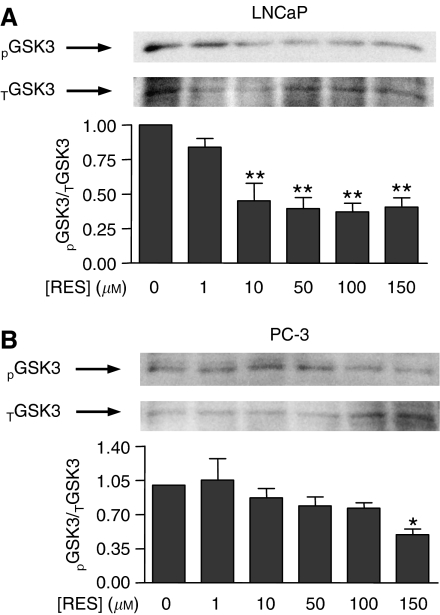
RES induces a pattern of GSK-3 phosphorylation in LNCaP and PC-3 cells that closely resembles that of PKB/AKT. LNCaP (**A**) and PC-3 (**B**) were treated with DMSO, 1, 10, 50, 100 or 150 *μ*M RES for 36 h in complete medium. Total cell extracts were analysed for phosphorylated (pGSK3) and total GSK-3 (tGSK3) by Western immunobloting using specific antibodies. Phospho-GSK-3 was normalised by total GSK-3 protein at each concentration of RES. Data shown are mean±s.e. from at least three different experiments. The differences are statistically significant with respect to control values (DMSO) at *P*<0.05 (^*^) or *P*<0.01 (^**^).

**Figure 7:**
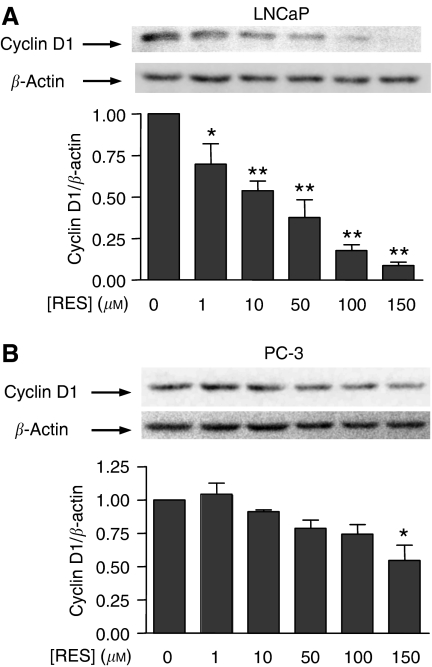
The GSK-3 target protein cyclin D1 is affected by RES in LNCaP and PC-3 cells with a pattern similar to that of PKB/AKT. LNCaP (**A**) and PC-3 (**B**) cells were left untreated (DMSO) or treated with 1, 10, 50, 100 or 150 *μ*M RES for 36 h in complete medium. Protein extracts were analysed for cyclin D1 levels by Western immunobloting using a specific antibody. The expression of *β*-actin was also determined to account for equal loading and protein quantitation. Cyclin D1 was normalised by *β*-actin and the results plotted for each concentration of RES. Data are shown as mean±s.e. from three different cultures. The differences with respect to control cultures (DMSO) are significant at *P*<0.05 (^*^) or *P*<0.01 (^**^).

One of best-characterised downstream targets of PI3K is PKB/AKT, which becomes activated by PDK1-dependent phosphorylation (Alessi *et al*, 1997; Engelman *et al*, 2006). Therefore, we next analysed if RES, through modulation of PI3K, could affect PKB/AKT and GSK-3 phosphorylation and cyclin D1 levels. After normalisation by total PKB/AKT protein, it was found that RES significantly decreased PKB/AKT phosphorylation in LNCaP cells, particularly at concentrations above 100 *μ*M (Figure 5A); in PC-3 cells, although PKB/AKT phosphorylation was also reduced, the effect was less pronounced (Figure 5B). Thus, inhibition of PI3K activity by RES resulted in a concentration-dependent decrease in PKB/AKT phosphorylation. Among the known targets of PKB/AKT, GSK-3 is a relevant signalling molecule controlling the level of cell cycle regulatory proteins. In agreement with the lower levels of PKB/AKT activation, RES also decreased GSK-3 phosphorylation in both LNCaP (Figure 6A) and PC-3 cells (Figure 6B). As GSK-3 becomes inactivated by phosphorylation, these results indicated that RES progressively turned GSK-3 to an activated (dephosphorylated) state. Again, this effect was apparently more pronounced in LNCaP than in PC-3 cells (Figure 6A and B). Endogenous cyclin D1 levels are controlled by GSK-3 through a mechanism by which the active kinase phosphorylates and targets cyclin D1 for proteasomal degradation. In agreement with increased GSK-3 activity, cyclin D1 protein content was reduced by RES in a concentration-dependent manner, and more strongly in LNCaP (Figure 7A) than in PC-3 cells (Figure 7B). These data strongly suggest that RES inhibited the AR- and ER*α*-associated PI3K activities in LNCaP and PC-3 cells and that they resulted in lower PKB/AKT activity, increased GSK-3 activation and decreased cyclin D1 protein levels.

### RES decreased PKB/AKT phosphorylation in primary cultures of human prostate tumours

**Figure 8:**
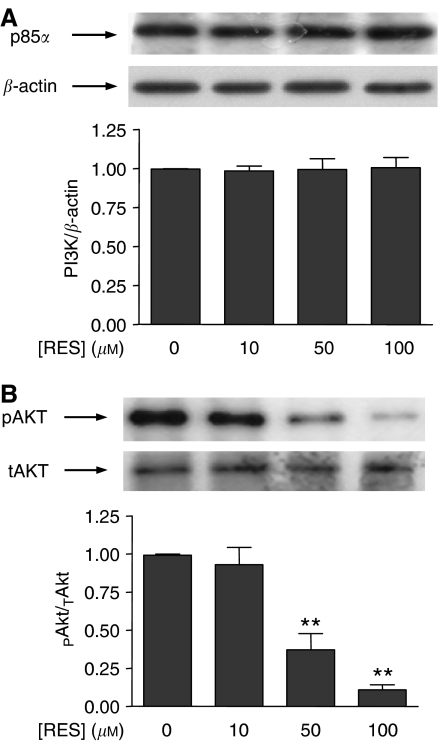
The effects of RES on p85/PI3K and PKB/AKT phosphorylation in cultured human primary prostate tumour cells closely resemble those observed in LNCaP and PC-3 cells. A total of seven biopsies from patients scheduled for radical prostatectomy were used to obtain primary cultures of prostate epithelial tumour cells. Cells were characterised as having a transformed phenotype as previously were indicated (Sanchez *et al*, 2005; Castellon *et al*, 2006, 2005). Cultures treated with DMSO, 10, 50 or 100 *μ*M RES for 36 h in complete medium. (**A**) Total protein extracts were analysed for p85/PI3K expression by Western immunobloting and the results normalised by *β*-actin levels. (**B**) Phosphorylated (pAKT) and total PKB/AKT (tAKT) were also determined by Western immunobloting and the amount of active protein (pAKT) normalised by the total PKB/AKT (tAKT). Data are shown as mean±s.e. The differences with respect to untreated control cultures are significant at *P*<0.01 (^**^).

To confirm and to further validate the results obtained in human prostate tumour cell lines LNCaP and PC-3, we have also determined the effect of RES on PKB/AKT phosphorylation in primary cultures from human prostate tumours. The transformed phenotype of these cultures was confirmed by immunocytochemistry for the marker PCTA-1 as described (Sanchez *et al*, 2005; Castellon *et al*, 2006, 2005). PKB/AKT was used as a reporter in the signalling pathway, as its degree of phosphorylation reflects the level of PI3K activity. RES did not significantly affect p85/PI3K protein levels (Figure 8A) in prostate tumour cells growing in primary culture, as previously observed for the cell lines analysed (see Figure 1). In primary cultures of prostate tumour cells, RES induced a concentration-dependent decrease in PKB/AKT phosphorylation that closely resembled that found in LNCaP and PC-3 cell lines (Figure 8B). Interestingly, a large degree of inhibition was observed at 100 *μ*M RES, indicating that these primary cells were very sensitive to this phytoalexin.

## DISCUSSION

Among the many different chemopreventive natural compounds identified to date, RES has been, and actually is, the focus of intense investigation. Its ability to inhibit growth and to induce apoptotic cell death in a large series of tumour cells, its potential to be easily included in the diet, and its activity as co-adjuvant for some chemotherapeutic molecules, make RES a good candidate for chemoprevention and chemotherapy of human cancer. Prostate cancer is a form of this disease with one of the highest prevalence and mortality in the population, having a poor prognosis once it becomes refractory to chemotherapy and/or metastatic. A relevant additional factor that contributes to the progression of prostate cancer and that limits treatment efficacy is the ability of tumour cells to change from a hormone-responsive to a hormone-unresponsive phenotype. In this context, the fact that RES has anti-oestrogenic activity in hormone-responsive tumour cells (Lu and Serrero, 1999; Bowers *et al*, 2000; Bhat *et al*, 2001; Levenson *et al*, 2003) opens the possibility for this molecule to be useful in prostate cancer. Indeed, different laboratories have reported antiproliferative activity of RES in hormone-responsive LNCaP (Hsieh and Wu, 1999; Mitchell *et al*, 1999; Narayanan *et al*, 2003) and hormone-unresponsive PC-3 and DU-145 prostate cells (Hsieh and Wu, 1999), albeit the former was more sensitive than the latter to cell death. This different potency of RES in prostate tumour cells could be related to downregulation of the expression and function of the AR in androgen-responsive LNCaP cells (Mitchell *et al*, 1999). Our data also support this different sensitivity of prostate tumour cells to RES, as the higher degree of cell death found in LNCaP with respect to PC-3 (Benitez *et al*, 2007) appears to correlate with a higher inhibition of PI3K activity in the former.

Non-nuclear functions have been described for steroid hormone receptors that emphasise their relevance in signalling pathways controlling proliferation and survival. In this context, androgens activated the PI3K pathway by inducing the interaction of AR with p85 (Sun *et al*, 2003), in a similar manner to that observed for the oestrogen-dependent activation of PI3K through ER*α* in endothelial cells (Simoncini *et al*, 2000). RES regulates the interaction between steroid hormone receptors and PI3K because concentrations of this molecule inducing apoptosis in MCF-7 also inhibited the ER*α*-dependent PI3K pathway (Pozo-Guisado *et al*, 2004), and a recent report has shown that RES inhibited PKB/AKT phosphorylation in LNCaP cells (Aziz *et al*, 2006).

In our experimental conditions, RES did not significantly affect protein levels of p85/PI3K in LNCaP or PC-3 cells, in contrast to a recent report showing a 40–50% reduction in p85 protein in LNCaP (Aziz *et al*, 2006). However, RES inhibited AR and ER*α* expression in LNCaP and PC-3 cells, respectively. The antiproliferative activity of RES in these cell lines (Benitez *et al*, 2007), if mediated through PI3K, would most probably produce a specific inhibition of the steroid receptor-associated PI3K (owing to the anti-oestrogenic potential of this molecule) than a global downregulation of cellular PI3K activity. Regardless the decrease in p85 expression, coimmunoprecipitation experiments revealed that none of the concentrations of RES used compromised AR- or ER*α* interaction with p85 in LNCaP or PC-3, indicating that inhibition of the steroid-receptor-dependent PI3K activity by RES was not due to changes in AR, ER*α* or p85 levels. In agreement, previous data showed that although RES decreased ER*α* protein levels in MCF-7, it did not affect the interaction between this receptor and p85/PI3K (Pozo-Guisado *et al*, 2004).

AR- and ER*α*-dependent PI3K activities were strongly inhibited by RES in LNCaP and PC-3 cells in a concentration dependent-manner in both, steroid-containing and steroid-depleted medium. As the interaction between AR and ER*α* with p85/PI3K was not affected by RES, the inhibition of PI3K in LNCaP and PC-3 was probably due to the anti-oestrogenic activity of this phytoalexin on both steroid receptors. It is interesting to note that short-term treatment (30 min) with high concentrations of RES significantly inhibited PI3K activity in LNCaP and PC-3 cells. Although in these conditions RES did not induce a significant degree of cell death in either cell line (D Benitez, unpublished observations), it could be possible that the inhibition of PI3K is an early step in the mechanism for apoptosis induction by RES in steroid-dependent tumour cells. Thus, the question remains of whether a maintained inhibition of steroid receptor-associated PI3K activity is required for RES-induced cell death or if, once triggered, apoptosis proceeds irreversibly in the absence of RES.

A major target of PI3K is PKB/AKT, which signals to downstream proteins such as GSK-3 (Cantley, 2002; Engelman *et al*, 2006). Consistent with the inhibition of PI3K activity by RES, PKB/AKT was inhibited, whereas GSK-3 was activated in a concentration-dependent manner in both LNCaP and PC-3. It is known that an increase in GSK-3 activity induces blockade of the cell cycle by promoting the degradation of G1/S regulators such as cyclin D1 (Scheid and Woodgett, 2001). Indeed, RES decreased cyclin D1 levels in either cell line, a result consistent with the ability of this phytoalexin to block DNA synthesis and to inhibit not only entry into S phase (Kuwajerwala *et al*, 2002) but also cell proliferation (Ratan *et al*, 2002). Interestingly, the effects of RES on PKB/AKT, GSK-3 and cyclin D1 were more pronounced in LNCaP than in PC-3, again suggesting that this molecule, although able to antagonise AR and ER*α* steroid receptors, could have a differential effectiveness on each one of them. Previous studies have also shown that RES inhibited more strongly cell growth and proliferation in LNCaP than in PC-3 (Hsieh and Wu, 1999). Further, inhibition of the PI3K signalling pathway by RES, at concentrations inducing apoptosis and through an ER*α*-dependent mechanism, has been also reported in MCF-7 tumour cells (Pozo-Guisado *et al*, 2004).

**Figure 9:**
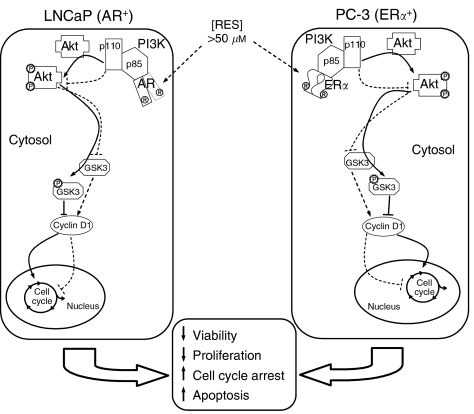
Proposed mechanism for RES induced apoptosis in LNCaP and PC-3 human prostate tumour cells. Concentrations of RES that induces apoptosis (RES>50 *μ*M) inhibits the AR- and ER*α*-dependent PI3K activity in LNCaP and PC-3 cells, respectively. RES-dependent PI3K inhibition results in lower levels of pPKB/AKT and in a decrease in its activity. Downstream PKB/AKT, hypophosphorylated GSK-3 would contribute to decreased cyclin D1 levels. Alteration of these pathways by RES would inhibit proliferation and increase apoptosis in an AR- or ER*α*-dependent manner. Filled lines represent signal transduction in absence of RES, whereas dotted lines stand for the effects of RES on the same signalling events.

A relevant aspect of our study is the analysis of primary cultures established from human prostate tumours biopsies. In close agreement to the results obtained in LNCaP and PC-3 cell lines, RES did not affect the expression of p85/PI3K in cultured human prostate tumour cells. Notably, however, RES induced a marked concentration-dependent inhibition of PKB/AKT phosphorylation. Therefore, RES inhibited the PI3K pathway in primary prostate tumour cells in culture through a mechanism similar to that found in cell lines. These observations support LNCaP and PC-3 as model cell lines in these studies and highlight the PI3K pathway as a potential target for the antiproliferative activity of RES in human prostate cancer. A proposed model for RES-dependent inhibition of the AR- and ER*α*-associated PI3K pathway is shown in Figure 9.

In summary, this work reveals that the AR- and ER*α*-dependent PI3K pathways are active in LNCaP and PC-3 human prostate tumour cells. Our data suggest that the antiproliferative activity of RES in androgen-responsive LNCaP and androgen-unresponsive PC-3 cells could be mediated, at least in part, by the antagonistic activity of this molecule on the AR and ER*α* that interact with the PI3K survival pathway. As it is plausible that the mechanism proposed here in tumour cell lines could also take place in human prostate tumours, we suggest that inhibition of steroid receptor-associated PI3K activity could represent a possible target for chemoprevention and for adjuvant chemotherapy involving RES. Additional *in vivo* studies and a detailed characterisation of this signalling pathway in human primary prostate tumours are required.
